# EEG Microstates in Mood and Anxiety Disorders: A Meta-analysis

**DOI:** 10.1007/s10548-023-00999-0

**Published:** 2023-08-24

**Authors:** Alina Chivu, Simona A. Pascal, Alena Damborská, Miralena I. Tomescu

**Affiliations:** 1 CINETic Center, National University of Theatre and Film “I.L. Caragiale” Bucharest, Bucharest, Romania; 2 Faculty of Psychology and Educational Sciences, Department of Applied Psychology and Psychotherapy, University of Bucharest, Bucharest, Romania; 3 Department of Psychiatry, Faculty of Medicine, Masaryk University and University Hospital Brno, Brno, Czech Republic; 4 Multimodal and Functional Neuroimaging Research Group, CEITEC – Central European Institute of Technology, Masaryk University, Brno, Czech Republic; 5 Faculty of Educational Sciences, Department of Psychology, University “Stefan cel Mare” of Suceava, Suceava, Romania; 6 Faculty of Psychology and Educational Sciences, Department of Cognitive Sciences, University of Bucharest, Bucharest, Romania

**Keywords:** Microstates, Meta-Analysis, Depression, Anxiety, PTSD, Bipolar Disorder, Panic Disorder

## Abstract

To reduce the psycho-social burden increasing attention has focused on brain abnormalities in the most prevalent and highly co-occurring neuropsychiatric disorders, such as mood and anxiety. However, high inter-study variability in these patients results in inconsistent and contradictory alterations in the fast temporal dynamics of large-scale networks as measured by EEG microstates. Thus, in this meta-analysis, we aim to investigate the consistency of these changes to better understand possible common neuro-dynamical mechanisms of these disorders.

In the systematic search, twelve studies investigating EEG microstate changes in participants with mood and anxiety disorders and individuals with subclinical depression were included in this meta-analysis, adding up to 787 participants.

The results suggest that EEG microstates consistently discriminate mood and anxiety impairments from the general population in patients and subclinical states. Specifically, we found a small significant effect size for B microstates in patients compared to healthy controls, with larger effect sizes for increased B presence in unmedicated patients with comorbidity. In a subgroup meta-analysis of ten mood disorder studies, microstate D showed a significant effect size for decreased presence. When investigating only the two anxiety disorder studies, we found a significantly small effect size for the increased microstate A and a medium effect size for decreased microstate E (one study). However, more studies are needed to elucidate whether these findings are diagnostic-specific markers.

Results are discussed in relation to the functional meaning of microstates and possible contribution to an explanatory mechanism of overlapping symptomatology of mood and anxiety disorders.

## Introduction

Mood and anxiety are the most common and debilitating disorders that frequently co-occur in individuals as concurrent diagnoses (Goldstein-Piekarski et al. 2016). For example, approximately 90% of anxiety patients will at least once in life experience a major depressive episode (Gorman, 1996). Moreover, the same pharmaceutical (antidepressants) and psychotherapeutic treatment interventions (cognitive-behavioral) are used to address the patient’s symptomatology across both disorders. Indeed, mood and anxiety disorders share highly overlapping symptoms such as tension, anxious arousal, anhedonia, melancholia, and normative mood (Grisanzio et al. 2018). In addition, depressed mood is accompanied by maladaptive spontaneous cognition with a shift towards ruminative, overfocused thoughts on negative scenarios and disrupted underlying resting-state functional networks (Chaieb et al. 2022). For example, in patients with a history of maltreatment and depressed mood, the results showed a reduction in positive thoughts and functional connectivity between the anterior cingulate cortex and a frontoparietal network related to attention and cognitive control (Hoffmann et al. 2018).

Common disrupted neural network circuitry, such as limbic structures like the anterior cingulate, amygdala, insula, and prefrontal cortices, are reported in mood and anxiety patients (Ressler and Mayberg 2007). More recently, disrupted connectivity in functional networks related to emotion dysregulation in anxiety disorders was suggested (Xu et al. 2019), as well as evidence of large-scale brain network dysfunction in mood disorders provided (Anand 2019; Kaiser et al. 2015). Transdiagnostic symptoms might be related to a specific large-scale brain network connectivity pattern. For example, hypo-connectivity during resting-state within the salience and attention resting-state networks might facilitate symptoms of anxious avoidance, negative emotional biases, and inattention/cognitive dyscontrol (Goldstein-Piekarski et al. 2022), and self-related negatively biased ruminative spontaneous cognition (Hoffman et al., 2018) – core overlapping symptoms that transcend both mood and anxiety disorders.

Fast-changing disruptions in spontaneous emotional regulation and ongoing brain activity are better identified by exploiting the sub-second temporal resolution of the electroencephalography (EEG) in quieter and more comfortable environments. Fast dynamics of large-scale functional brain network activity are captured using the microstate analysis (Pascual-Marqui et al. 1995). Microstates are periods of quasi-stable spatial configurations of scalp-recorded EEG potentials lasting around 100 ms (Lehmann et al. 1987). Between four (termed A-D) and seven (termed A-G), microstates have been described, and converging evidence of multiple studies suggest that microstates are related to the well-known resting state functional network such as the salience, dorsolateral attention, and default-mode networks (Michel and Koenig 2018). In other words, the synchronized activity of large-scale functional brain networks is reflected in temporal parameters of EEG microstates, such as coverage, occurrence and mean duration (Michel and Koenig 2018). Time coverage is a temporal parameter representing the percentage of time a microstate was active. Microstate occurrence represents how many times a certain microstate was present per second, independent of how long a microstate lasts, the information quantified by the mean duration parameter. Changes in these microstate temporal dynamic quantifiers have been linked to various cognitive states, mental disorders, and change as a function of pharmacological or psycho-social interventions (Khanna et al. 2015; Linton et al. 2022; Michel and Koenig 2018; Schiller et al. 2019; Tomescu et al. 2022). Microstates investigation in the clinical population shows differences in mood disorders (Bissonnette et al. 2022; Chen et al. 2022; Damborská et al. 2019a, b; He et al. 2021; Murphy et al. 2020; Sun et al. 2022; Wang et al. 2021), including subclinical populations (Qin et al., 2022; Xue et al. 2021; Zhao et al. 2022) and anxiety disorders such as panic disorder (PD) (Kikuchi et al. 2011) and post-traumatic stress disorder (PTSD) (Terpou et al. 2022). The reported changes seem to affect all microstates with high inter-study variability. Furthermore, evidence on microstate differences across mood disorders is contradictory. For example, both null, increase, and decrease of microstate D have been reported in the literature on both mood and anxiety disorder patients (Bissonnette et al. 2022; Chen et al. 2022; Damborská et al. 2019a, b; He et al. 2021; Murphy et al. 2020; Sun et al. 2022; Wang et al. 2021). Microstate D is among the canonical microstates and is systematically associated with the dorsal attention network (Michel and Koenig 2018). It might be essential to elucidate if such a temporal disrupted activity is consistent across these patients. Generally, many patients report attention and executive functioning impairment.

We conducted a meta-analysis of these publications to establish the consistency of evidence of disrupted microstate temporal dynamics in people suffering from highly co-occurring mood and anxiety disorders. Elucidating possible confounding effects of medication, comorbidity with other conditions, year of publication, and gender prevalence, we believe the results of this study will advance future research on identifying objective biomarkers that may contribute to improvements in diagnostics and treatment efficacy of most common mental health disorders.

## Methods

### Literature Search

A systematic literature search was conducted in Web of Science, Pubmed, and Scopus to identify potentially relevant studies for this meta-analysis. The investigation was completed in December 2022, with an update in February 2023. The keywords used were: “EEG microstate” “anxiety”, “depression”, “bipolar”, “PTSD”, and based on the PICO criteria (Spring 2007), the following algorithm was defined: (eeg microstate) AND (anxi* OR depres* OR bipolar OR ptsd).

### Selection Criteria

Articles for this meta-analysis were selected according to the inclusion and exclusion criteria initially established. Based on the general objective of the study and after consulting the relevant literature on the above criteria, the following inclusion criteria were considered: (a) articles written in English, (b) the participants must be adults or adolescents, (c) the presence of at least four microstate classes (A, B, C, D), (d) presence of a patient group and a control group, (e) resting-state condition, (f) sufficient data to calculate effect sizes (means and standard deviations), (g) patients with anxiety disorders, mood disorders or subclinical depression. Exclusion criteria were the following: (a) articles published in a language other than English, (b) the study population did not target adolescents or adults, (c) fewer than four microstates’ classes were studied, (d) no control group, (e) participants were completing experimental tasks, (f) there were insufficient data to calculate effect sizes, (g) patients with mental disorders other than anxiety disorders, depressive disorders, PTSD, or bipolar disorder.

### Selection Process

**Fig. 1:**
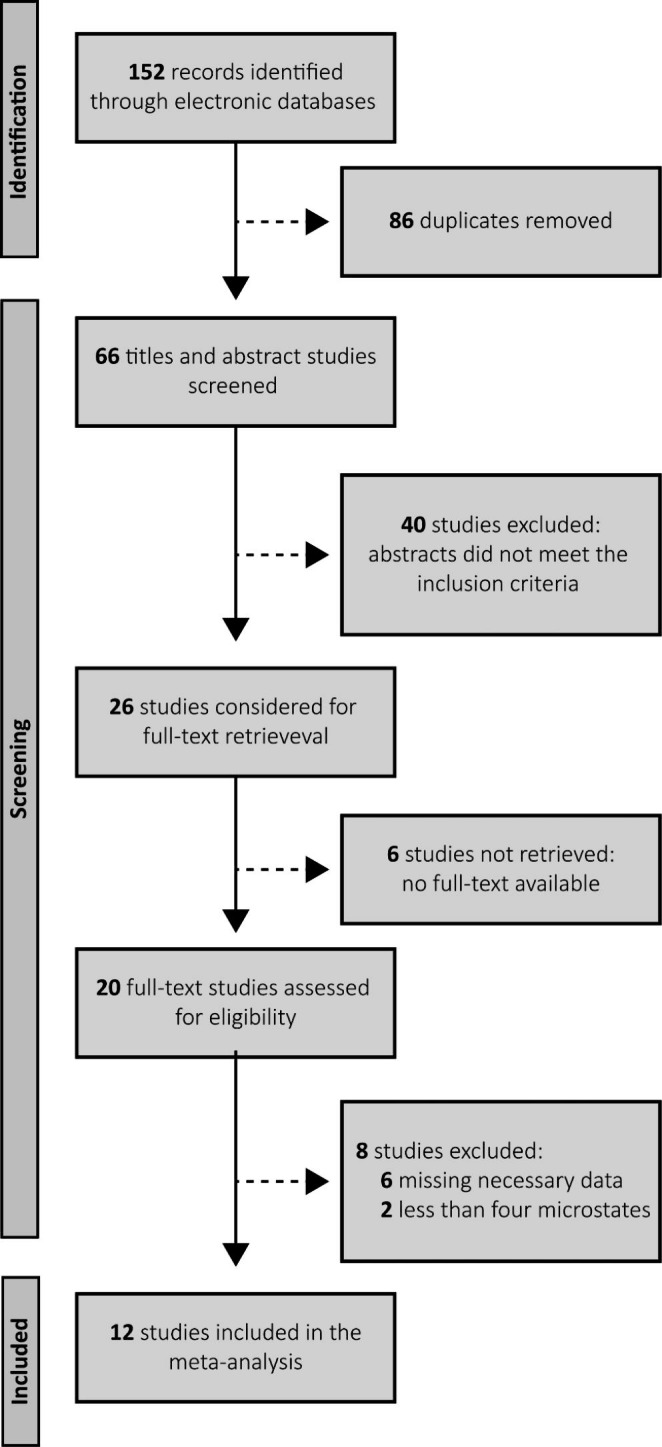
PRISMA diagram describing the study selection process

The systematic database search initially identified 152 articles. We eliminated 86 duplicates of these, leaving 66 articles available for screening. Based on the title and information available in the abstract, we eliminated 40 articles that matched the exclusion criteria. The full-text search for 26 articles resulted in 20 for final eligibility screening. In addition, we excluded six articles for insufficient data for analysis, and two did not contain microstate class data. In the end, 12 articles matched the inclusion criteria for the meta-analysis. Figure 1 shows the PRISMA diagram (Page et al. 2021) with the selection process for the studies included in this meta-analysis.

Among these twelve articles, one includes two data sets (Murphy et al. 2020). One data set was with patients with major depressive disorder and a control group; the second was with patients with remitted major depressive disorder and a control group. Therefore, we included them as separate data sets.

### Included Studies

Analyzing the data sets of 12 selected studies, we found that the sample sizes ranged from 34 to 142, resulting in 787 participants. Regarding age group, 11 studies included adults, and one study (He et al. 2021) had children/adolescents. The mean age ranged from 14.58 to 53 years. Regarding gender, the proportion of female participants also ranged from 29.41 to 100%.

Related to the clinical status, we included studies with the following clinical status: five major depressive disorder (MDD) patient studies (Bissonnette et al. 2022; Damborská et al. 2019b; He et al. 2021; Murphy et al. 2020; Sun et al. 2022), one study investigating MDD patients in remission (rMDD) (Murphy et al. 2020), two publications on subclinical MDD (subMDD, high risk HR-MMD, low risk LR-MDD) participants (Qin et al. 2022; Zhao et al. 2022), four studies with bipolar disorder (BD) patients (Chen et al. 2022; Damborská et al. 2019a, b; Wang et al. 2021), one study with panic disorder (PD) (Kikuchi et al. 2011), and one study with posttraumatic stress disorder (PTSD) (Terpou et al. 2022), while four studies assessed depressive symptoms as the main feature (Bissonnette et al. 2022; Damborská et al. 2019b; He et al. 2021; Murphy et al. 2020). In addition, ten studies included a clinical sample (Bissonnette et al. 2022; Chen et al. 2022; Damborská et al. 2019a, b; He et al. 2021; Kikuchi et al. 2011; Murphy et al. 2020; Sun et al. 2022; Terpou et al. 2022; Wang et al. 2021), and two presented data from a subclinical selection (Qin et al. 2022; Zhao et al. 2022). Regarding comorbidities, two articles reported that patients suffered from psychotic symptoms and temporal lobe epilepsy (TLE) (Sun et al. 2022; Wang et al. 2021). The characteristics of the included studies are shown in Table 1.

**Table 1:** Characteristics of included studies

Reference	N		Mean age		% W	Groups (and *comorbidity)*	Medication
	P	HC	P	HC			
Bissonnette et al. (2022)	14	21	32.36	41.43	65	MDD vs. HC	ns
Chen et al. (2022)	19	16	42.40	35.50	62	BD vs. HC	Ap, Ad, MS
Damborská et al. (2019a)	17	17	35.90	36.60	29	Euthymic BD vs. HC	Ap, Ad, MS
Damborská et al. (2019b)	19	19	53.00	51.40	31	MDD BD vs. HC	Be, Ap, Ad, MS
He et al. (2021)	35	35	14.58	15.05	64	MDD vs. HC	-
Kikuchi et al. (2011)	18	18	30.20	30.60	38	PD vs. HC	-
Murphy et al. (2020)	63	79	29.20	27.50	70	MDD vs. HC	Psy (N = 10/63)
Murphy et al. (2020)	30	79	32.70	27.50	73	rMDD vs. HC	-
Qin et al. (2022)	34	34	20	ns	61	HR-MDD vs. LR-MDD	-
Sun et al. (2022)	19	19	26.00	29.00	35	MDD (+ *TLE*) vs. TLE	-
Terpou et al. (2022)	61	61	41.51	40.90	68	PTSD vs. HC	Ad, aAp, Se, St
Wang et al. (2021)	26	35	22.80	24.90	62	BD (+ *PS*) vs. HC	MS, aAp,
Zhao et al. (2022)	40	38	18.51	18.72	100	subMDD vs. HC	-

ns – Unspecified, *TLE* – Temporal lobe epilepsy comorbidity, *PS* – Psychotic symptoms comorbidity, Ap -Antipsychotics, Ad -Antidepressants, MS- Mood stabilizers, Be- Benzodiazepine, Psy-Psychotropic, aAp-Atypical Antipsychotics, Se-Sedatives, St-Stimulants

### Moderators and Extracted Data

A data coding system describes the characteristics of each study, as shown in Table 1. The characteristics considered are author references, year of article publication, the sample size for each group, the average age of participants in each group, percentage of the gender of participants, the composition of each group in terms of mood and anxiety disorders, comorbidities, and medication treatment of participants.

Five relevant moderators were considered in this study: mean age, gender percentage, and year of publication as continuous moderators and medication and comorbidity as categorical moderators.

Age and gender were selected as moderators since the literature demonstrates significant differences across different ages, and between men and women in the duration and occurrence of microstates (Koenig et al., 2002, Tomescu et al. 2018).

The year of publication is another essential moderator to consider. Over the years, methodological standards have changed and may impact data quality. Although most articles included in this meta-analysis were published in recent years, this analysis may highlight the differences between older and recent studies.

Regarding medication, studies indicate that data on microstate characteristics differ in patients treated with medication (Kikuchi et al. 2007). Therefore, considering medication as the moderator may help explain the effects obtained.

Anxiety disorders are highly comorbid with mood disorders (Johansson et al. 2013) and strongly influence each other. By including the comorbidity moderator, we aim to investigate the extent to which comorbid conditions may affect the effect found.

### Statistical Analysis

Statistical data analysis was performed using Comprehensive Meta-Analysis (CMA) software, version 2.2.064 (Borenstein et al. 2005). The data extracted for analysis were the mean and standard deviation for both conditions (experimental and control groups). We extracted the microstate temporal parameters of mean duration (ms) and occurrence (Hz) as these are independent temporal parameters the most often reported in the literature. For the main objective of this study, the random effects model was chosen as the type of meta-analysis.

The Hedges’ *g* indicator was selected to estimate the effect sizes. A value of Hedges’ *g* between 0.20 and 0.50 indicates a small effect size, a value between 0.50 and 0.80 indicates a medium effect size, whereas a value of at least 0.80 indicates a large effect size (Cohen 1988). P-values were adjusted for multiple comparisons using the false discovery rate (FDR) correction (p < 0.05) (Benjamini and Hochberg 1995).

For moderator analyses, we used subgroup difference analysis for the categorical predictors (comorbidity and medication), and the meta-regression procedure was used for the continuous ones (year of publication, gender percentage, and age) (Borenstein et al. 2009). As Qin et al.‘s article (2022) did not provide missing info on mean age of experimental group, it was excluded from the moderation analysis on age.

## Results

### Mood and Anxiety disorders - subMDD, MDD, rMDD, BD, PTSD, and PN

**Fig. 2:**
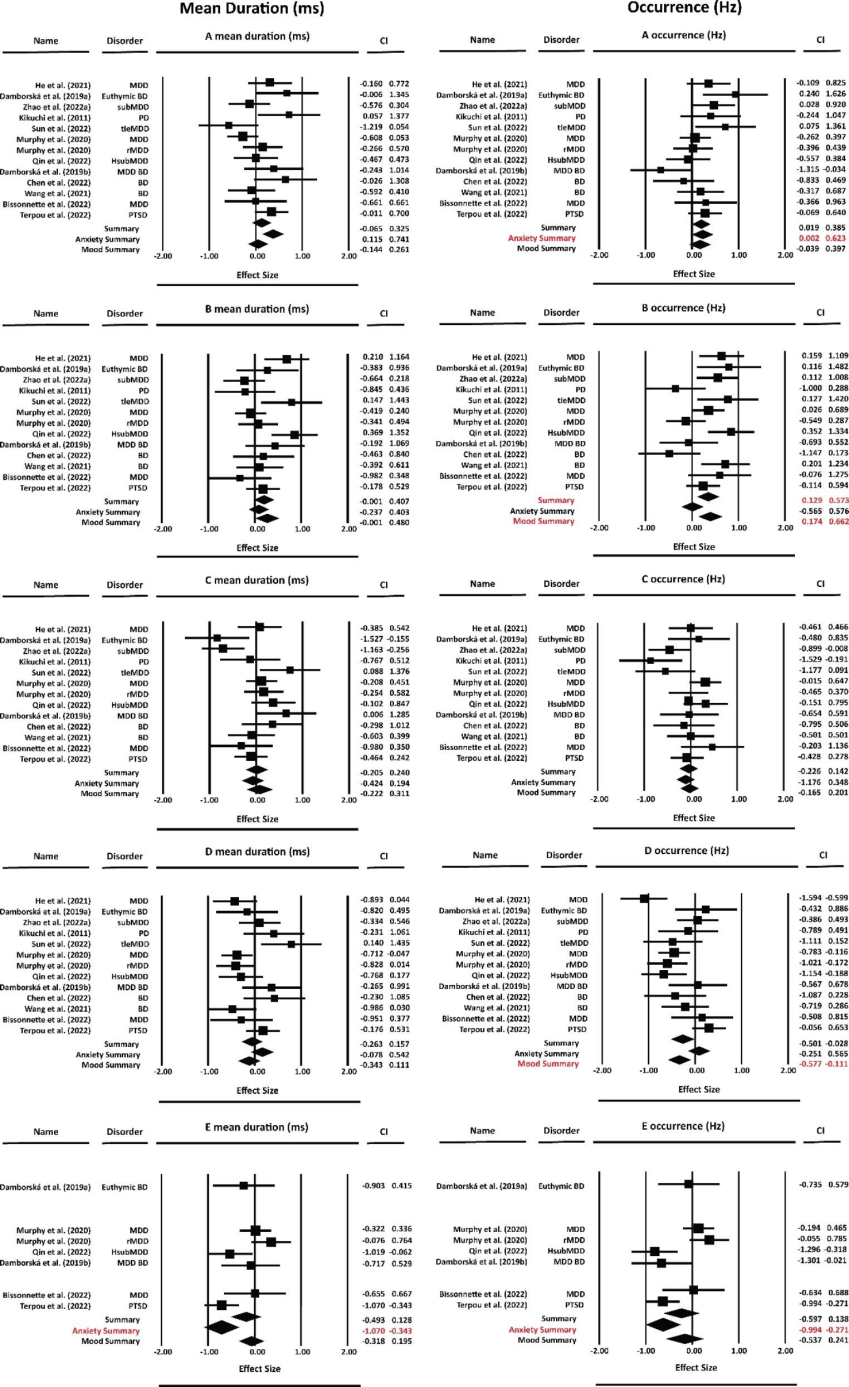
Forest plots of meta-analysis results for each microstate and temporal parameter. Left side - *mean duration*, right side - *occurrence*; Hedges’ *g* (squares proportional to weights used in meta-analysis) and associated confidence intervals (lateral tips) for individual data sets; summary measures (diamonds) of the whole group, anxiety subgroup that included two studies (Kikuchi et al. 2011; Terpou et al. 2022), and the mood subgroup that consists of ten studies (Bissonnette et al. 2022; Chen et al. 2022; Damborská et al. 2019a, b; He et al. 2021; Murphy et al. 2020; Qin et al. 2022; Sun et al. 2022; Wang et al. 2021; Zhao et al. 2022)

Using a random-effects model, we found a significant and small effect size in patients vs. HC comparison for microstates B *occurrence*, see Table 2. Microstate B occurred significantly more in patients (Fig. 2).

**Table 2:** Microstates meta-analysis results

**Mean duration (ms)**					
Microstates	**A**	**B**	**C**	**D**	**E**
**Mood and Anxiety disorders**					
N	13	13	13	13	7
*g*	0.13	0.2	0.01	-0.05	-0.18
95% CI	-0.06 to 0.32	-0.001 to 0.4	-0.2to 0.24	-0.26 to 0.15	-0.49 to -0.12
*p*	0.41	0.13	0.9	0.752	0.46
**Mood disorders**					
N	11	11	11	11	6
*g*	0.05	0.23	0.04	-0.11	-0.06
95% CI	-0.14 to 0.26	-0.00 to 0.48	-0.22 to 0.31	-0.34 to 0.11	-0.31 to 0.19
*p*	0.75	0.13	0.82	0.525	0.752
**Anxiety disorders**					
N	2	2	2	2	**1**
*g*	**0.42**	0.08	-0.11	0.23	**-0.7**
95% CI	**0.11 to 0.74**	-0.23 to 0.4	-0.42 to 0.19	-0.07 to 0.54	**-1.07 to -0.34**
*p*	**0.03**	0.75	0.66	0.32	**p < 0.000**
**Occurrence (Hz)**					
Microstates	**A**	**B**	**C**	**D**	**E**
**Mood and Anxiety disorders**					
N	13	**13**	13	13	7
*g*	0.2	**0.35**	-0.04	-0.26	-0.22
95% CI	0.01 to 0.38	**0.12 to 0.57**	0.22 to 0.14	-0.5 to 0.02	-0.59 to 0.13
*p*	0.11	**0.015**	0.75	0.116	0.44
**Mood disorders**					
N	11	**11**	11	**11**	6
*g*	0.17	**0.41**	0.01	**-0.34**	-0.14
95% CI	-0.03 to 0.39	**0.17 to 0.66**	-0.16 to 0.2	**-0.57 to -0.11**	-0.53 to 0.24
*p*	0.27	**0.01**	0.9	**0.024**	0.66
**Anxiety disorders**					
N	2	2	2	2	**1**
*g*	0.31	0.005	-0.41	-0.15	**-0.63**
95% CI	0.00 to 0.62	-0.56 to 0.57	-1.17 to 0.34	-0.25 to 0.56	**-0.99 to -0.27**
p	0.13	0.98	0.5	0.66	**0.01**

N = number of data sets, *g* = Hedges’ g, CI = confidence interval, *p* = FDR corrected p-values

### Mood disorders - subMDD, MDD, rMDD, BD

When performing the meta-analysis only for the studies on patients with mood disorders, there was a significant small effect size for microstate B and microstate D *occurrence*. More specifically, microstate B appears more frequently, and microstate D occurs significantly less in mood disorder patients when compared to healthy individuals (Fig. 2).

### Anxiety disorders - PTSD and PD

When performing the meta-analysis only for the studies on patients with anxiety disorders, we found a significant small effect size for microstate A and a medium effect size for microstate E *mean duration* (Table 2). In terms of *occurrence*, the results showed a small significant effect for microstate A and a medium effect size for class E. These results imply that microstate A tends to occur more frequently and has a longer duration. In contrast, microstate E occurs less frequently and with a shorter duration in people with anxiety disorders when compared with healthy controls.

### Medication and Comorbidity Moderation

To investigate how medication and comorbidity in people with mood and anxiety disorders might have influenced the results of this meta-analysis, we performed a moderation analysis.

The moderation results for comorbidity revealed significant effect sizes for microstate B *occurrence* (*g* = 0.443, 95% CI [0.046 to 0.841], *p* = 0.02, FDR corrected). In addition, we found a more significant effect size for B *occurrence* in mood patients with comorbidity studies (*g* = 0.742) than in mood patients without comorbidity (*g* = 0.387).

Mood and anxiety patient’s medication moderation analysis revealed significant effect sizes for the *occurrence* of microstate B (*g* = 0.371, 95% CI [0.112 to 0.630], *p* = 0.05, FDR corrected) with larger effect sizes for unmedicated mood and anxiety patients (*g* = 0.518) than medicated patients (*g* = 0.303).

### Age, Gender, and year of Publication Meta-regression

To see if the year of publication, age and gender are implicated in the effect of anxiety and depressive symptoms on EEG microstates, we performed moderation analyses for each microstate.

In the case of continuous moderators, the meta-regression results revealed non-significant associations related to age (β = 0.0004, 95% CI [-0.004 to 0.005], *p* = 0.884), the year of publication (β = 0.003, 95% CI [-0.018 to 0.023], *p* = 0.783) and gender percentage (β = -0.003, 95% CI [-0.005 to 0.00007], *p* = 0.057). These results might also be considered as a trend for gender percentage moderation where decreased microstate presence was observed in studies with more women. However, results should be considered with care as possibly driven by studies including mostly men (29% women - Damborská et al. (2019a) or only women Zhao et al. (2022).

### Publication Bias

**Fig. 3:**
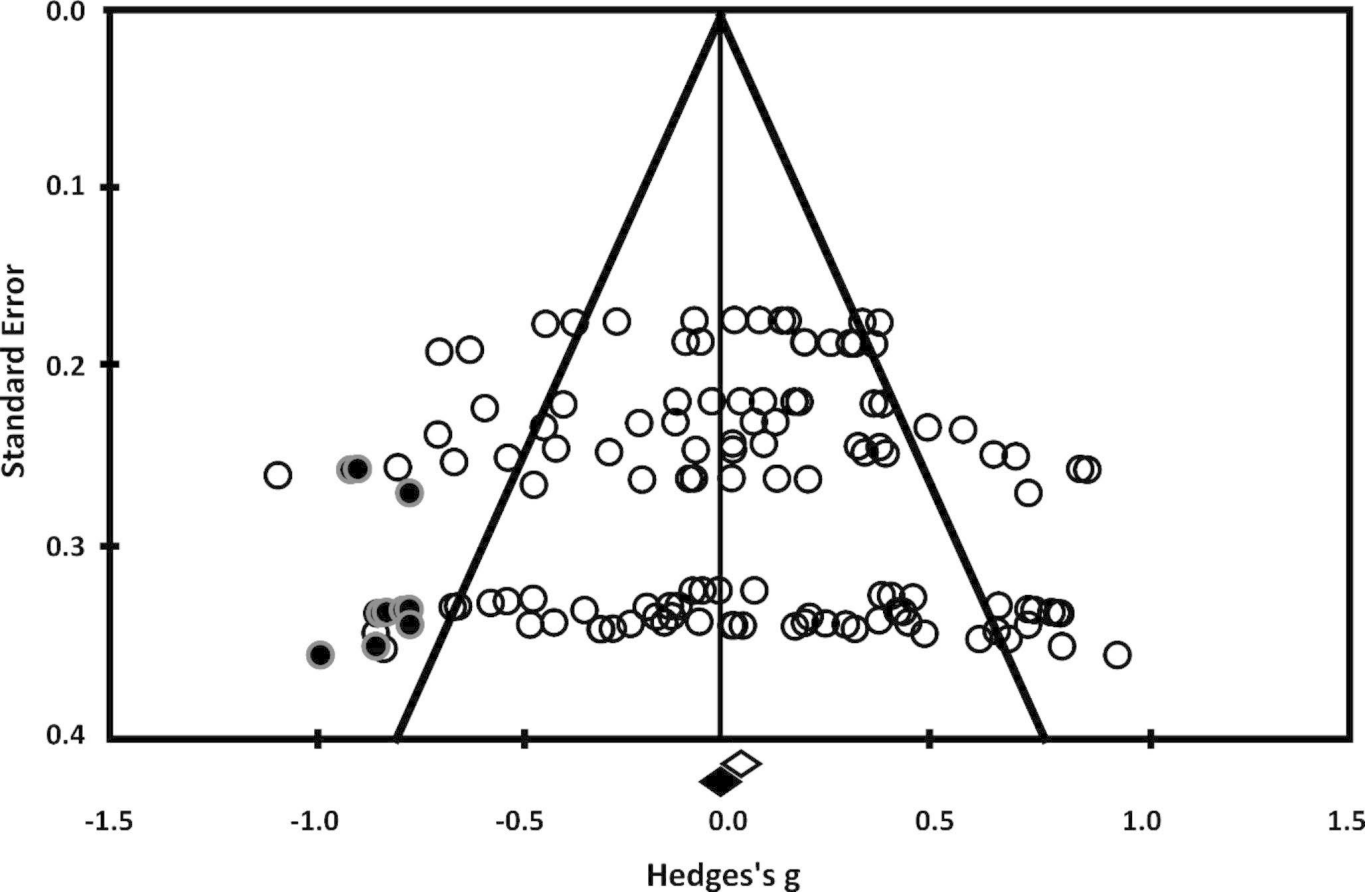
The funnel plot of publication bias and standard error associated with Hedges’s *g* precision in the data of the twelve studies included in the meta-analysis. Black dots indicate inputted missing studies needed for the symmetrical plot

Finally, we investigated the publication bias in the studies included in the meta-analysis. By visually inspecting the skewed funnel plot, precisely the standard error for each study’s effect size, we can observe a slight publication bias on the left side of the figure (Fig. 3). Given these results, we used the following two methods to analyze this publication bias in more detail.

To obtain corrected effect sizes and confidence intervals of the relationship between the effect size and the associated variance, we used Duval & Tweedie trim and fill procedure (Duval and Tweedie 2000; Higgins and Green 2011). This method first eliminates studies that might be responsible for the skewness of the distribution. Then, it estimates the true center of the funnel by replacing the missing studies and their missing pair. The results assessed 11 studies with an effect size (g = -0.031, 95% CI [-0.11 to 0.05]) lower than the mean of the initial results (g = 0.036, 95% CI [-0.040 to 0.112], p = 0.358), so the effect size would be adjusted. Thus, we used Egger’s intercept test (Egger et al. 1997); Higgins and Green 2011), which showed a symmetrical funnel plot (intercept = 0.64; 95% CI [–0.58 to 1.86]) Fig. 3. However, it is necessary to keep in mind that the significance thresholds associated with these indicators are limited by the small number of studies considered in this meta-analysis.

## Discussion

EEG microstates have been identified as possible biomarkers across multiple mental health disorders and treatment interventions. We systematically investigated whether microstates consistently differ with mood and anxiety disorders across twelve published studies. Using the meta-analytic approach, mood and anxiety disorder patients show a significant pattern of increased presence of B microstates (Fig. 2; Table 2). The separate mood and anxiety disorder meta-analyses indicate that decreased D microstate presence might be specific to mood disorders (Fig. 2; Table 2). In contrast, anxiety disorder patients mainly presented increased A and decreased E presence (Fig. 2; Table 2).

These effects could not be explained by year of publication, age and gender, as moderator analysis did not reach the significance threshold. However, comorbidities such as temporal lobe epilepsy and psychotic symptoms (Table 1) predicted the occurrence of B microstates (increased in both mood and anxiety patients, and mood patients only). In addition, patients with comorbidity seemed to have larger effect sizes than those without comorbidity. However, these results should be carefully interpreted as only two out of twelve studies presented more than one diagnosis (Table 1). Moreover, previous studies on patients with psychotic symptoms mostly showed decreased microstate B presence (Rieger et al. 2016).

Microstate B occurred more and with larger effect sizes in studies with unmedicated patients suggesting that B might be associated with the clinical state and might support possible compensatory mechanisms in unmedicated patients. Indeed, microstate B temporal dynamics were positively related to the intensity of depressive symptomatology as measured by self-reported scales such as the BDI (Atluri et al. 2018; Yan et al. 2021). Microstate B is most often associated with bottom-up networks and visual activity in the left-right cuneus, inferior, and middle occipital gyrus (Britz et al. 2010; Custo et al. 2017; Michel and Koenig 2018). Interestingly, the activity of the B microstate was previously reported to increase after visual stimulations and during eyes-open resting states, and within engagement in visually auto-biographical memory recollection (Bréchet et al. 2019; D’Croz-Baron et al. 2021; Seitzman et al. 2017; Tarailis et al. 2023). In light of these findings, our results suggest that mood and anxiety disorder patients might engage too often in visually related past experiences such as rumination symptomatology, which fail to compensate for the mood and anxiety symptoms and negatively impact mental health. Indeed, microstate B presence was positively associated with self-related thoughts about self-behavior and feelings (Zanesco et al. 2020) and depression symptomatology as measured by self-reported scales such as the BDI (Atluri et al. 2018; Yan et al. 2021). In parallel, B microstates are negatively associated with spontaneous thoughts about the future and problem-solving (Zanesco et al. 2020), mental activity involving more high-order cognitive networks like dorsal attention.

Microstate D has been associated with the dorsal attention network and functional activity (increased EEG source activity/negative BOLD activity) in the frontal and parietal cortices’ right-lateralized dorsal and ventral areas using both EEG and EEG-fMRI methods (Bréchet et al., 2019, Britz et al. 2010; Custo et al. 2017; Yuan et al. 2012). It is among the most frequently observed canonical four microstates and has a distinctive spatial scalp distribution with a central - to right posterior local maxima (Michel and Koenig 2018). In this meta-analysis, microstate’s D decreased *occurrence* was a consistent finding across the ten studies on mood disorder patients. Murphy et al. 2020 suggested that this disrupted dynamic might be related to a vulnerability trait marker as both MDD patients in remission and high-risk individuals show decreased microstate D dynamic (Murphy et al. 2020; Qin et al. 2022). Moreover, MDD patients had a significant negative correlation with depressive symptomatology, where higher symptomatology is predicted by lower D *occurrence* (Murphy et al. 2020; Qin et al. 2022). Furthermore, inflammatory markers, such as interleukin-2, tumor necrosis factor-α, and C-reactive protein that are higher in primary depression patients, negatively correlate with microstate D *occurrence* (Zhao et al. 2022). Microstate D disruption might be a vulnerability trait marker that could help identify at-risk individuals and benefit from earlier interventions and better treatment. Only a few studies investigated treatment response as a function of D modulations. However, different types of interventions seem to have a beneficial effect on symptomatology and D occurrence. These studies investigated the treatment response of electroconvulsive and pharmacology therapy. Results show that significant modulations of D *occurrence* were associated with decreased symptom severity as measured by the BDI scale (Atluri et al. 2018; Lei et al. 2022). In addition, a feasibility study on microstate neurofeedback intervention showed that healthy individuals successfully upregulated their microstate D occurrence (Diaz Hernandez et al. 2016). The authors explored the possibility of neurofeedback training on microstate D upregulation as a possible treatment for schizophrenia and high-risk individuals (Diaz Hernandez et al. 2016). Indeed, microstate D decreased occurrence was a consistent finding in schizophrenia patients and individuals at high genetic risk, including healthy relatives (da Cruz et al. 2020; Rieger et al. 2016; Tomescu et al. 2014, 2015). These results might suggest microstate D could be considered as a transdiagnostic marker possibly associated with the overlapping depressed mood and negative symptomatology present among the schizophrenia patients, such as anhedonia, for example. An alternative explanation might relate to the overlapping observed cognitive impairment across both disorders, such as attention and executive functioning impairment.

A one-to-one relation of EEG microstates with functional resting-state networks should be made with caution, studies investigating cognitive state modulations on temporal dynamics of microstates partially support the view that microstate D is associated with the dorsal attention network involving allocation and maintenance of attentional resources (Tarailis et al. 2023). In addition, microstate D quantifiers positively correlate with alertness and reaction time scores in a non-clinical population (Zanesco et al. 2020). While some studies report a decreased presence during states of visualization or verbalization (Antonova et al. 2022; Milz et al. 2016), other studies report that microstate D is more present when participants are asked to perform demanding cognitive tasks, such as mental serial subtraction tasks based on focused states of attention (Seitzman et al. 2017, Bréchet et al., 2019). Microstate D might be related to attention and cognitive control deficits only during attention-demanding tasks. Alternatively, the contradictory results might also be explained by possible overlapping temporal dynamics between C, D and E microstates when forcing the number of states to the canonical four microstates (Custo et al. 2017; Tarailis et al. 2023, Michel and Koenig 2018). Some studies might suggest the association between D and attention network should be considered with care as negative BOLD might reflect deactivation rather than activation of the dorsal attention network (Antonova et al. 2022). More importantly, microstate D is less present in socially induced spontaneous relaxed states (Tomescu et al. 2022) and shows reduced presence with altered states of attention, consciousness, and lack of cognitive control, such as during auditory-verbal hallucinations in SZ patients (Kindler et al. 2011), deep hypnosis (Katayama et al. 2007), sleep, and dreaming (Bréchet et al. 2020; Brodbeck et al. 2012).

Our findings align with a recent brain model of depression that involves the disruption of functional and effective connectivity among the high-order networks, including the dorsal attention networks (Li et al. 2018). These impaired temporal dynamics of the visual-episodic memory-related B microstate and dorsal-attention network-related D microstate might be responsible for over-engagement in past-oriented negative episodic events and emotions and failure of top-down cognitive control in mood disorder patients.

Thus, future studies should address how these modulations predict behavior and cognitive functioning in mood disorder patients. In addition, investigations on larger clinical populations should clarify if microstate D occurrence might act as a transdiagnostic marker of psychopathology, as several studies, including schizophrenia patients, report evidence of D occurrence decreased presence (Michel and Koenig 2018; Rieger et al. 2016; Tomescu et al. 2014, 2015).

When looking at only the anxiety disorder patients that suffer from PD and PTSD, we observed a pattern of brain network dynamic of increased microstate A and decreased microstate E presence (small to medium effect sizes, respectively). Microstate A is one of the four canonically reported microstates related to neuronal activity in the bilateral superior, middle temporal lobe, auditory, and language processing cortices (Britz et al. 2010; Custo et al. 2017; Michel and Koenig 2018). However, during the cognitive task manipulation, microstate A is more engaged during visual activity and eyes open resting (Milz et al. 2016; Seitzman et al. 2017), suggesting that microstate A might be associated with a less specialized sensory network. Microstate E was associated with dorsal anterior cingulate, inferior frontal gyrus, and insular cortices (Custo et al. 2017). The salience network, including regions of the anterior cingulate and insular cortices regions, was previously related to the microstate C activity (Britz et al. 2010). E is among the less canonical but highly reproducible microstates in studies that objectively identified an optimal number of states instead of a canonical selection of four (A-D) microstates. Please see the review on this topic for a relevant discussion on objectively determining the number of microstates (Michel and Koenig 2018). A more relevant finding in Custo et al. (2017), is that C and E microstates might overlap in their temporal dynamics when opting for the canonical four. In our meta-analysis, more than half of the included studies report microstate E across mood and anxiety disorders. Results are significant (moderate effect size) only when examining anxiety disorders. Terpou et al. (2022) proposed that the brain regions functionally related to the salience network and decreased E microstates might reflect a failure to map relevant bottom-up stimuli resulting in a hypervigilance state in patients suffering from anxiety-related disorders like PTSD (Terpou et al. 2022). However, more studies on anxiety disorder patients that make available the necessary data (means and standard deviations of microstates parameters for both groups) are needed to elucidate if increased A and decreased E microstate is a specific pattern of microstate dysregulation in anxiety disorders.

Taken together, the results of this meta-analysis point toward an over-engagement of microstates B and A related to bottom-up, visual, and auditory-language networks and under-engagement of D and E microstates associated with top-down attention/cognitive control and salience network in mood and anxiety patients. These disrupted dynamics might sustain a possible explanatory mechanism by which, due to trait-related failures of cognitive control and salience mapping in mood and anxiety disorders, patients might engage in over-compensatory states of increased spontaneous thoughts of visual-auditory/language, possibly self-related, autobiographical memories that are biased toward failure and negative outcome scenarios.
