# Correction: EEG Microstates in Mood and Anxiety Disorders: A Meta-analysis

**DOI:** 10.1007/s10548-023-01028-w

**Published:** 2023-12-09

**Authors:** Alina Chivu, Simona A. Pascal, Alena Damborská, Miralena I. Tomescu

**Affiliations:** 1 CINETic Center, National University of Theatre and Film “I.L. Caragiale” Bucharest, Bucharest, Romania; 2 Faculty of Psychology and Educational Sciences, Department of Applied Psychology and Psychotherapy, University of Bucharest, Bucharest, Romania; 3 Department of Psychiatry, Faculty of Medicine, Masaryk University and University Hospital Brno, Brno, Czech Republic; 4 Multimodal and Functional Neuroimaging Research Group, CEITEC – Central European Institute of Technology, Masaryk University, Brno, Czech Republic; 5 Faculty of Educational Sciences, Department of Psychology, University “Stefan cel Mare” of Suceava, Suceava, Romania; 6 Faculty of Psychology and Educational Sciences, Department of Cognitive Sciences, University of Bucharest, Bucharest, Romania

The article “EEG Microstates in Mood and Anxiety Disorders: A Meta-analysis”, written by Alina Chivu, Simona A. Pascal, Alena Damborská, Miralena I. Tomescu was originally published electronically on the publisher’s internet portal on 24 August 2023 without open access. With the author(s)’ decision to opt for Open Choice the copyright of the article changed on 5 December 2023 to © The Author(s) 2023 and the article is forthwith distributed under a Creative Commons Attribution 4.0 International License, which permits use, sharing, adaptation, distribution and reproduction in any medium or format, as long as you give appropriate credit to the original author(s) and the source, provide a link to the Creative Commons licence, and indicate if changes were made. The images or other third party material in this article are included in the article’s Creative Commons licence, unless indicated otherwise in a credit line to the material. If material is not included in the article’s Creative Commons licence and your intended use is not permitted by statutory regulation or exceeds the permitted use, you will need to obtain permission directly from the copyright holder. To view a copy of this licence, visit http://creativecommons.org/licenses/by/4.0

